# Optimizing genome editing efficiency in wheat: Effects of heat treatments and different promoters for single guide RNA expression

**DOI:** 10.5511/plantbiotechnology.23.0717a

**Published:** 2023-09-25

**Authors:** Mitsuko Kishi-Kaboshi, Fumitaka Abe, Yoko Kamiya, Kanako Kawaura, Hiroshi Hisano, Kazuhiro Sato

**Affiliations:** 1 Institute of Crop Science, National Agriculture and Food Research Organization (NARO), Tsukuba, Ibaraki 305-8518, Japan; 2 Kihara Institute for Biological Research, Yokohama City University, Yokohama, Kanagawa 244-0813, Japan; 3 Institute of Plant Science and Resources, Okayama University, Kurashiki, Okayama 710-0046, Japan

**Keywords:** CRISPR/Cas9, genome editing, promoter, *Triticum aestivum* L.

## Abstract

Genome editing is a promising method for simultaneously mutagenizing homoeologs in the three subgenomes of wheat (*Triticum aestivum* L.). However, the mutation rate via genome editing must be improved in order to analyze gene function and to quickly modify agronomic traits in wheat. Here, we examined the Cas9-induced mutation rates in wheat plants using two promoters for single guide RNA (sgRNA) expression and applying heat treatment during *Agrobacterium tumefaciens*-mediated transformation. Using the *TaU6* promoter instead of the *OsU6* promoter from rice (*Oryza sativa* L.) to drive sgRNA expression greatly improved the Cas9-induced mutation rate. Moreover, a heat treatment of 30°C for 1 day during tissue culture increased the Cas9-induced mutation rate and the variety of mutations obtained compared to tissue culture at the normal temperature (25°C). The same heat treatment did not affect the regeneration rates of transgenic plants but tended to increase the number of transgene integration sites in each transgenic plant. These results lay the foundation for improving the Cas9-induced mutation rate in wheat to enhance research on gene function and crop improvement.

## Introduction

Bread wheat (*Triticum aestivum*, 2*n*=6*x*=42; AABBDD) is a hexaploid species with a very large genome (17 Gb) consisting of three closely related A, B, and D subgenomes (IWGSC 2014). Modifying specific traits in wheat via random mutagenesis is challenging and laborious because in most cases, all homoeologous copies of a gene must be inactivated before a phenotype can be observed. However, using current genome editing techniques, it is now possible to target multiple genes at once, including homoeologs, making genome editing a major strategy for systemically introducing mutations in wheat and other polyploid crops. Editing of the wheat genome has been successfully used to target genes underlying important agronomic traits, such as pathogen resistance (Brauer et al. 2020; Li et al. 2022; Wang et al. 2014; Zhang et al. 2017), starch properties (Li et al. 2021), and seed dormancy (Abe et al. 2019).

Whole-genome sequencing has also facilitated genome editing of wheat. A fully annotated reference genome of the wheat cultivar ‘Chinese Spring’ was recently released (IWGSC 2018), followed by the construction of high-quality genome assemblies for multiple wheat lines (Walkowiak et al. 2020). This available whole-genome information enables the genetic analysis of agronomic traits in wheat by facilitating the identification of wheat genes that are orthologous to those with desirable functions in other plant species. Notably, many agronomic traits are determined by multiple genetic factors, which requires the genetic modification of multiple genes to generate agronomically useful varieties via genome editing. We recently generated a chromosome-level genome assembly of the transformation-amenable wheat cultivar ‘Fielder’ (Sato et al. 2021); this assembly might aid in the design of genome editing constructs with specific target sequences while avoiding off-target effects.

Clustered regularly interspaced short palindromic repeats (CRISPR)/CRISPR-associated protein 9 (Cas9)-mediated gene editing is widely used for genome editing. In the CRISPR/Cas9 system, a ribonucleoprotein complex comprising the Cas9 nuclease and a single guide RNA (sgRNA) performs sgRNA-directed cleavage of the genomic DNA sequence complementary to the sgRNA. The resulting double-stranded break is then repaired by the DNA repair machinery, which occasionally introduces a mutation in the DNA sequence. To deploy CRISPR/Cas9 systems in plants, Cas9 and at least one specific sgRNA must be introduced into the plant cell, which is often accomplished by Agrobacterium (*Agrobacterium tumefaciens*)-mediated transformation of a construct expressing these factors. Indeed, Agrobacterium-mediated transformation has many advantages over other available methods and allows for the stable introduction of intact (not truncated) transgenes with a low copy number in the host genome, as demonstrated in rice (*Oryza sativa*) (Dai et al. 2001; Hiei et al. 1994; Jeon et al. 2000) and barley (*Hordeum vulgare*) (Travella et al. 2005). By contrast, transformation of the plant genome via the biolistic-aided delivery of transgenes can result in the integration of numerous vector-derived fragments in the genome (Liu et al. 2019; Svitashev et al. 2002) and produces plants with lower fertility compared to those generated by Agrobacterium-mediated transformation (Dai et al. 2001). Agrobacterium-mediated transformation is therefore the method of choice for obtaining genome-edited plants.

Agrobacterium-mediated wheat transformation has been successfully employed in the cultivar ‘Fielder’ (Ishida et al. 2015). However, whereas Agrobacterium-mediated transformation is easy using the rice cultivar ‘Nipponbare’ or the Arabidopsis (*Arabidopsis thaliana*) accession Columbia-0, wheat transformation is laborious and difficult and achieves only low efficiency, even when using ‘Fielder’. It takes approximately four months to grow wheat plants suitable for obtaining explants for transformation. These explants are immature embryos collected at 14–17 days post anthesis, which must be carefully isolated from surface-sterilized seeds under a stereomicroscope in a clean bench. Following inoculation with Agrobacterium, it takes four months to regenerate plants. In light of these requirements, it is important to increase the transformation efficiency and Cas9-induced mutation rate of wheat. However, the transformation efficiency is affected by numerous factors, such as plant growth conditions, the developmental stages of the embryos, treatments during tissue culture, and culture conditions. These factors make it difficult to perform wheat transformation and genome editing on a routine basis. Even if transformants are obtained, not all carry mutations of interest. Furthermore, in order to mutate all homeologous genes in the wheat subgenomes simultaneously, the genome editing efficiency must be improved.

To increase the efficiency of genome editing, it is also important to focus on the vector components and culture conditions. The promoter that drives the expression of the sgRNA cassette is a critical parameter that determines the efficiency of Cas9-induced mutation, as it dictates the level, timing, location, and tissue specificity of transgene expression. The small nucleolar RNA U3 and the small nuclear RNA U6 are non-coding RNAs that function in the nucleus. The expression of *U3* and *U6* in plants is driven by RNA polymerase III, and their promoters are considered to be suitable for expressing sgRNAs. The *OsU3* and *OsU6* promoters from rice and the *AtU6-26* promoter from Arabidopsis are commonly used to drive sgRNA expressions in plants (Feng et al. 2013; Jiang et al. 2013; Shan et al. 2013). However, using a *U3* or *U6* promoter from the species being transformed to drive sgRNA expression yielded higher genome editing efficiencies compared to the use of the *OsU6* or *AtU6* promoter in soybean (*Glycine max*), sorghum (*Sorghum bicolor*), grape (*Vitis vinifera*), and cotton (*Gossypium hirsutum*) (Long et al. 2018; Massel et al. 2022; Ren et al. 2021; Sun et al. 2015). In wheat, we and another group showed that the use of the *TaU6* promoter resulted in a higher Cas9-induced mutation rate per transgenic plant compared to using the *OsU6* promoter (Kamiya et al. 2020; Liu et al. 2020).

Temperature is another important parameter that affects genome editing efficiency in plants. Temperatures above that for typical plant growth increase the mutation rate in transgenic plants harboring the CRISPR/Cas9 system in Arabidopsis, sweet orange (*Citrus*×*sinensis*), and wheat (Kurokawa et al. 2021; LeBlanc et al. 2018; Milner et al. 2020). One possible reason for the increased Cas9-induced mutation rate is the higher activity of the Cas9 nuclease at higher temperature, as demonstrated by growing Arabidopsis in vitro at 37°C or 22°C, the latter being the common growth temperature for Arabidopsis in a laboratory setting (Kurokawa et al. 2021; LeBlanc et al. 2018).

In this study, we evaluated the effects of using different promoters to drive sgRNA expression and the effects of heat treatments on the Cas9-induced mutation rate to improve the genome editing efficiency of wheat via Agrobacterium-mediated transformation. We present an optimized protocol that employs the wheat *TaU6* promoter and heat treatment and provide fundamental information for further improving the efficiency of genome editing in wheat.

## Materials and methods

### Plant materials and growth conditions

The spring wheat (*Triticum aestivum* L.) cultivar ‘Fielder’ was used for analysis. For transformation, seeds were sown in plastic pots containing a 2 : 1 (v/v) mixture of Sakata Supermix A (fine peat moss, Sakata Seed): Nippi fertilized granulated soil (Nihon Hiryo), and 0.1% (v/v) controlled-release fertilizer (Osmocote Exact Mini 3-4M 15-9-11+2MgO+TE, Hyponex Japan). Plants were grown in a glasshouse with day/night temperatures of 16°C/10°C under an 8-h light/16-h dark photoperiod for 10–12 weeks. Just before the heading stage, the plants were transferred to a controlled environmental chamber with day/night temperatures of 20°C/13°C under a 14-h light (300–500 µmol m-^2^ s-^1^)/10-h dark photoperiod. Spikes were collected at 14–17 days after anthesis for transformation.

### Plasmid construction

**Figure 1:**
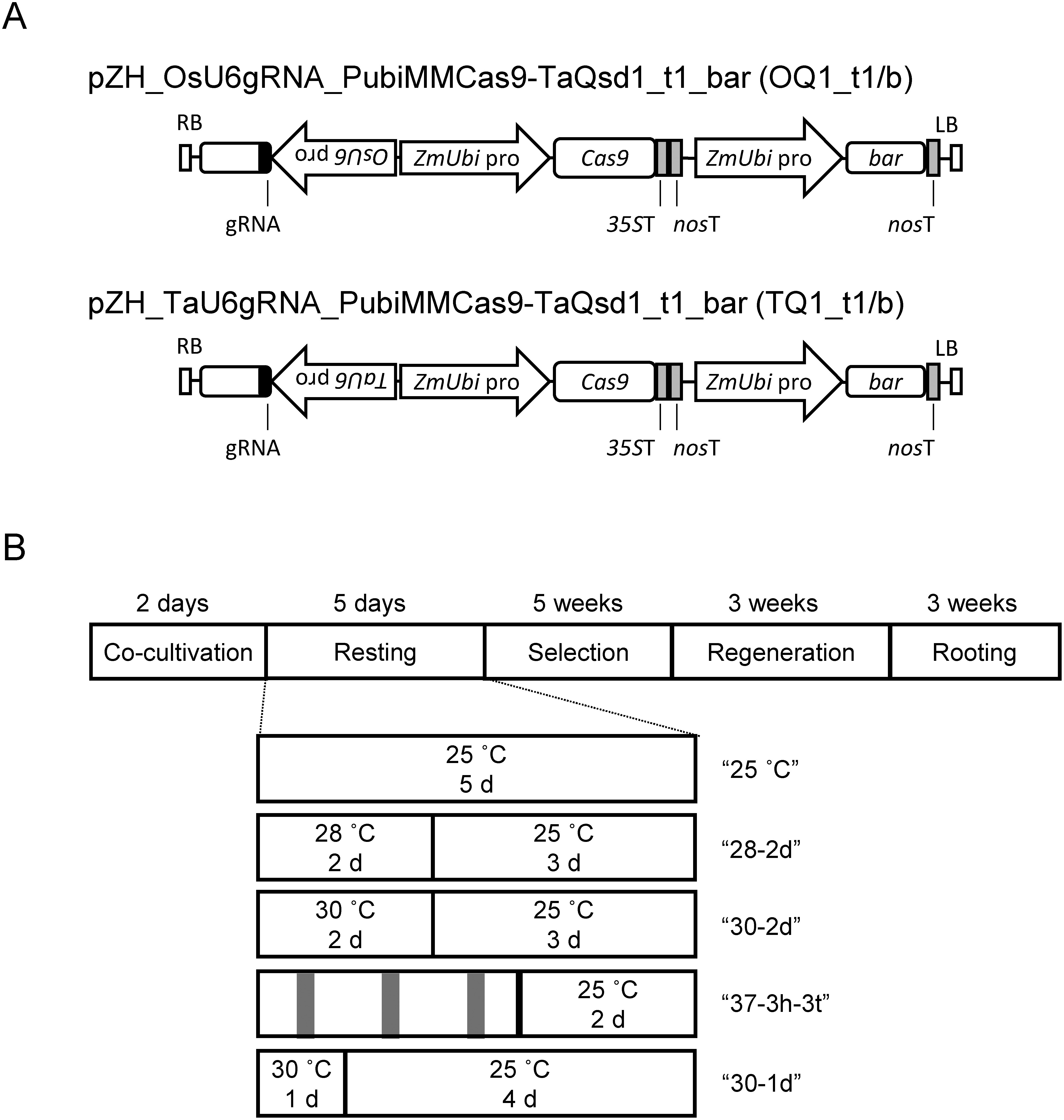
Figure 1. Assessment of the individual contributions of different promoters and heat treatments on transformation and genome editing efficiencies in wheat. (A) Diagrams of the two binary vectors used in this study. (B) Diagram of the transformation protocols following the co-cultivation of wheat embryos with Agrobacterium cultures without or with heat treatment during the resting step. Gray vertical bars in “37-3h-3t” indicate the timing of the 37°C treatments. *35S*T, cauliflower mosaic virus 35S terminator; *bar*, *bialaphos resistance gene*; LB, left border; *nos*T, *nopaline synthase* terminator; *OsU6* pro, *U6* promoter from *Oryza sativa*; RB, right border; *TaU6* pro, *U6* promoter from *Triticum aestivum*; *ZmUbi* pro, *Zea mays Ubiquitin* promoter.

The plasmids pZH_OsU6gRNA_PubiMMCas9-TaQsd1_t1_bar and pZH_TaU6gRNA_PUbiMMCas9-TaQsd1_t1_bar (Figure 1A) were constructed as follows. PrimeStar MAX DNA polymerase (Takara Bio) was used to amplify the various fragments for plasmid construction. Specifically, a DNA fragment containing the *Ubiquitin* promoter from maize (*Zea mays*) (*ZmUbi* pro), the *bialaphos resistance gene* (*bar*), and the *nos* terminator (*nos*T) was amplified by PCR from the pUBA vector (Toki et al. 1992). The plasmid backbone DNA was amplified by PCR using pZH_gYSA_PubiMMCas9 (Mikami et al. 2015) as a template. The amplified fragments were ligated using an In-Fusion HD PCR Cloning Kit (Takara Bio), yielding the pZH_gYSA_PubiMMCas9_bar plasmid. Two single guide RNA (sgRNA) expression cassettes, the *OsU6*pro:*TaQsd1*_t1:polyT and the *TaU6*pro:*TaQsd1*_t1:polyT DNA fragments, were excised from the pOsU6_TaQsd1_t1 and pTaU6_TaQsd1_t1 vectors (Kamiya et al. 2020) and cloned into pZH_gYSA_PubiMMCas9_bar using the restriction enzymes AscI and PacI. The oligonucleotide primers used are listed in Supplementary Table S1.

### Transformation

The explant used for transformation was immature embryos. Immature embryos were carefully isolated from surface-sterilized immature seeds under a stereomicroscope in a clean bench and inoculated with Agrobacterium (*Agrobacterium tumefaciens*) harboring either the pZH_OsU6gRNA_PubiMMCas9-TaQsd1_t1_bar or pZH_TaU6gRNA_PubiMMCas9-TaQsd1_t1_bar plasmid. Inoculation with Agrobacterium and generation of transgenic wheat were performed as described previously (Abe et al. 2020). Following Agrobacterium inoculation, co-cultivation, and removal of the embryo axis from each immature embryo, the remaining scutellum was transferred to resting medium. In the normal resting step, the scutella were incubated at 25°C for 5 days in the dark (Figure 1B). For heat treatment, the scutella were incubated at 28°C, 30°C, or 37°C and returned to 25°C in the dark until the end of the resting step (Figure 1B). During the selection, regeneration, and rooting steps, 5 mg l^−1^ phosphinothricin was used for selection instead of hygromycin, which was used in the previous report (Abe et al. 2020).

The regenerated T_0_ plants were transferred to soil and grown at 20°C/13°C under a 14-h light/10-h dark photoperiod in a controlled environmental chamber. When a spike emerged, it was bagged to allow self-propagation. The resulting T_1_ seeds were sown in plastic pots filled with soil and grown in a growth chamber for 10 days. To obtain F_1_ seeds, a wild type ‘Fielder’ spike with its anthers removed was bagged with a spike of T_0_ plants.

### RNA analysis

For RNA extraction, ten scutella were collected as one sample, immediately frozen in liquid nitrogen, and stored at −80°C. Total RNA was extracted from the sample using RNAiso (Takara), treated with DNase I (Qiagen), and further purified using an RNeasy Plant Mini kit (Qiagen). PrimeScript RT reagent with gDNA eraser (Perfect Realtime) (Takara) was used to synthesize cDNA from 1.0 µg of total RNA. PCR was performed using KOD One PCR Master Mix (Toyobo) with specific primers for the *Actin* and *Cas9* genes.

### PCR and sequence analysis

Genomic DNA was extracted from the leaf tips of regenerated T_0_ plants or T_1_ seedlings and used for PCR with Gflex DNA polymerase (Takara Bio). Transgenes were detected by amplifying the *Cas9* or *bar* gene. The mutation rate in the target sites of the *TaQsd1* gene was analyzed by cleaved amplified polymorphic sequence (CAPS) analysis. In the CAPS analysis, the *TaQsd1*-derived PCR amplicons were digested with the restriction enzyme PstI and subjected to agarose gel electrophoresis to detect the presence of the mutation. To detect the mutation, common primers for *TaQsd1* in the three subgenomes were used. Mutations in *TaQsd1* in each subgenome were detected using subgenome-specific primers. To analyze the sequences of candidate off-target sites, nested PCR was performed to amplify specific sequences. The *TaQsd1* sequences and off-target candidate sequences were analyzed using a capillary DNA analyzer. All oligonucleotide sequences are listed in Supplementary Table S1.

## Results and discussion

### Optimization of the experimental conditions: Preliminary experiments

To examine the effects of different promoters driving the sgRNA cassette on genome editing efficiency, we generated two plasmids, pZH_TaU6gRNA_PubiMMCas9-TaQsd1_t1_bar (TQ1_t1/b) harboring the *TaU6* promoter (*TaU6* pro) and pZH_OsU6gRNA_PubiMMCas9-TaQsd1_t1_bar (OQ1_t1/b) harboring the *OsU6* promoter (*OsU6* pro), to drive TaQsd1_t1 sgRNA in transgenic wheat (Figure 1A). The Agrobacterium-mediated wheat transformation procedure consisted of five steps: 1) co-cultivation with Agrobacterium, 2) a resting step in the presence of an antibiotic to remove the Agrobacteria, 3) selection with a selective agent to obtain transformed callus, 4) regeneration, and 5) rooting (Figure 1B). We did not detect chimeric mutant plants in our previous genome editing experiment (Abe et al. 2019), suggesting that any mutations should occur at an early time point during transformation.

The activity of the Cas9 nuclease is higher at 37°C than at 22°C in vitro (Kurokawa et al. 2021), which inspired us to test several heat treatments during the resting step in an attempt to increase the Cas9-induced mutation rate. First, we performed transformation experiments with heat treatment at 28°C or 30°C for 2 days (“28-2d” and “30-2d”, respectively; Figure 1B). We also tested a 37°C treatment for 3 h each day for the first 3 days (“37-3h-3t”) during the resting step, as we reasoned that continuous exposure to high temperature would damage wheat cells. Following heat treatment, we returned the calli to 25°C for further culture until the end of the resting period and proceeded with the subsequent steps, culminating with the regeneration of transgenic plants.

We cultivated one transgenic plant per callus peace even when more than two plants were regenerated from the same piece of callus. We then assessed the regenerated shoots for the presence of the transgene by genomic PCR and for mutations of *TaQsd1* in PCR-positive regenerated plants (transformants) by CAPS analysis (Supplementary Table S2, Supplementary Figure S1, Table 1). To detect mutations, we performed CAPS analyses of all *TaQsd1* copies using common primers among the three *TaQsd1* homoeologs. If a mutation was detected, we performed CAPS analysis of each *TaQsd1* homoeolog.

**Table 1:** Table 1. Summary of the preliminary transformation experiments, number of transgenic plants, and mutations of *TaQsd1*.

Heat treatment	sgRNA promoter	Number of experiments	Number of embryos transformed	Transgenic plants (number, rate)*	Genome-edited plants (number, rate)	Mutant genotype and number of plants**
30°C, 2 day (30-2d)	*OsU6*	1	50	0, 0%	0, 0%	NA, 0
	*TaU6*	1	60	4, 6.67%	2, 3.33%	*AaBBDD*, 1 *Aabbdd*, 1
28°C, 2 day (28-2d)	*OsU6*	4	249	7, 2.81%	1, 0.40%	*AABbDD*, 1
	*TaU6*	4	271	10, 3.69%	4, 1.48%	*AABbDD*, 1 *aabbdd*, 3
37°C, 3 h/d×3 (37-3h-3t)	*OsU6*	8	567	4, 0.71%	2, 0.35%	*AabbDD*, 1 *aabbdd*, 1
	*TaU6*	8	554	4, 0.72%	1, 0.18%	*AaBbDd*, 1

*The rate was calculated based on the number of regenerated PCR-positive transformants per callus piece. Only one regenerated plant per callus piece was retained. The *Cas9* or *bar* DNA fragment was detected by PCR. **TaQsd1 genotype and number of mutant plants. NA, not applicable.

Both the regeneration rates of transformants and the Cas9-induced mutation rates appeared to be higher when using the TQ1_t1/b vector vs. the OQ1_t1/b vector under both “28-2d” and “30-2d” conditions. We obtained regenerated plants in one out of two experiments under “30-2d” conditions and in seven out of eight experiments under “28-2d” conditions. Under “37-3h-3t” conditions, we obtained no regenerated plant in ten out of 16 experiments (Supplementary Table S2), and both the regeneration and Cas9-induced mutation rates were low. The results from these preliminary experiments indicate that using the *TaU6* promoter with a heat treatment of 28–30°C during the resting step is effective for mutagenesis using the CRISPR/Cas9 system via our transformation protocol.

### Heat treatment at 30°C increases the genome editing mutation rate and the rate of variation of mutated sequences

We repeated our experiments to investigate the effects of heat treatment at the resting step and different sgRNA promoters on the production of genome-edited wheat plants. In the preliminary experiment using “28-2d” and “30-2d” conditions, the Cas9-induced mutation rate was higher at “30-2d”, but we were concerned about the stability of callus regeneration following a long exposure to higher temperature. We therefore exposed immature wheat embryos to heat treatment of 30°C for 1 day (“30-1d”) before returning them to the normal temperature, or only treated the embryos with the normal temperature (“25°C”), each with either the OQ1_t1/b or TQ1_t1/b vector (Figure 1B). There was no obvious difference in the appearance of immature embryos or the *Cas9* expression level between the two conditions (Supplementary Figure S2). We performed eight experiments per condition to equalize the variation in materials and environmental conditions in each experiment (Supplementary Table S3, Table 2). The transformant regeneration rates were similar across all conditions (2.12–2.31%). These results indicate that the choice of sgRNA promoter and the “30-1d” heat treatment did not affect the transformant regeneration rate.

**Table 2:** Table 2. Summary of the transformation experiments, number of transgenic plants, and mutations of *TaQsd1.*

Heat treatment	sgRNA promoter	Number of experiments	Number of embryos	Transgenic plants (number, rate)*	Genome-edited plants (number, rate)	Mutant genotype** and plant line number
“25°C” (25°C, 5 day)	*OsU6*	8	567	12, 2.12%	0, 0%	
	*TaU6*	8	586	13, 2.22%	5, 0.85%	*AABBDd*, T7 *AABbDD*, T18 *AaBbDd*, T19 *aabbdd*, T12, T26
“30-1d” (30°C, 1 day)	*OsU6*	8	603	14, 2.32%	1, 0.17%	*AABbDD*, O20
	*TaU6*	8	585	13, 2.22%	6, 1.03%	*AABbDd*, T15 *AaBbdd*, T2 *aabbdd*, T10, T16, T23, T24

*The rate was calculated based on the number of regenerated PCR-positive transformants per callus piece. Only one regenerated plant per callus piece was retained. The *Cas9* or *bar* DNA fragment was detected by PCR. **TaQsd1 genotype.

We then examined the mutation rates at *TaQsd1* in each transformant by CAPS analysis (Supplementary Figure S3). Using the OQ1_t1/b vector, we detected zero (for “25°C”) and one (“30-1d”) transformant with a mutation in the *TaQsd1* gene. The single OQ1_t1/b transformant under “30-1d” conditions carried one mutation in one of the six copies of *TaQsd1* in the genome (with two copies for each homoeolog). By contrast, we obtained five and six transformants with one to six mutations in *TaQsd1* under “25°C” and “30-1d” conditions, respectively, using the TQ1_t1/b vector. Under the “25°C” condition, two T_0_ plants contained a mutation in one *TaQsd1* copy, and two T_0_ plants carried a mutation in all six *TaQsd1* copies. Under the “30-1d” condition using the TQ1_t1/b vector, we obtained one T_0_ plant with a mutation in two *TaQsd1* copies, one T_0_ plant with a mutation in four *TaQsd1* copies, and four T_0_ plants with a mutation in all six *TaQsd1* copies. These results indicate that the TQ1_t1/b vector is more effective than the OQ1_t1/b vector, while the “30-1d” condition increased the number of genome-edited plants and mutation sites per edited genome compared to the “25°C” condition. Moreover, the Cas9-induced mutation rate per embryo increased using the *TaU6* promoter.

**Figure 2:**
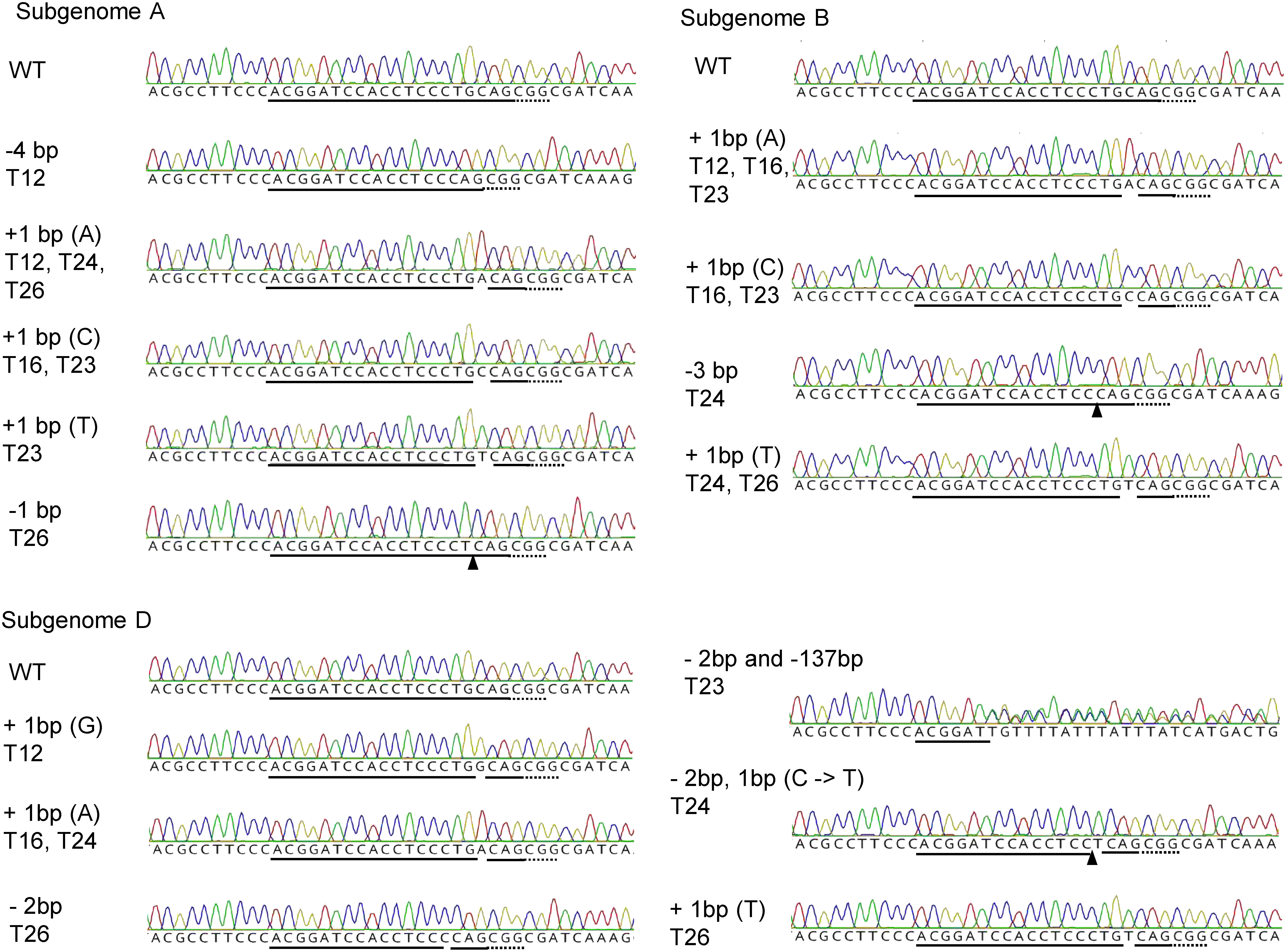
Figure 2. Summary of the mutant sequences of the genome editing target site in *TaQsd1*. Sequencing chromatograms of subgenome-specific TaQsd1 DNA fragments surrounding the sgRNA target site. DNA samples were obtained from T_1_ seedlings from T_0_ plants carrying a mutation in all six copies of *TaQsd1*. Solid lines indicate sequences corresponding to the sgRNA sequence. Triangles indicate the positions of deletions. Dotted lines indicate the positions of the PAM sequence.

The increased number of mutation sites per edited genome raised the possibility that heat treatment increased the rate of sequence variation at the mutation sites. We thus analyzed the sequence of each *TaQsd1* copy using T_1_ seedlings T12, T16, T23, T24, and T26, whose parents have the *aabbdd* genotype (Figure 2, Supplementary Table S3). There were 3 and 5 mutation types under the “25°C” condition and 5 to 6 mutation types under the “30-1d” condition using the TQ1_t1/b vector. These results suggest that heat treatment increased the sequence variation of the mutation sites.

The increase in mutation frequency raised the possibility of off-target mutations. We therefore searched for candidate off-target sites of TaQsd1_t1 sgRNA using the WheatCrispr website (Cram et al. 2019, https://crispr.bioinfo.nrc.ca/WheatCrispr/ (Accessed May 13, 2023)) and analyzed the sequence with the highest Cutting Frequency Determination (CFD) score (0.55) as a measure of sgRNA:DNA interactions (Supplementary Figure S4). There were no mutations in the above candidate off-target site of TaQsd1_t1. These results indicate that increasing the Cas9-induced mutation rate using the *TaU6* promoter and heat treatment did not result in increased off-target editing by TaQsd1_t1 sgRNA.

The above results indicate that heat treatment at 28–30°C during the resting step strongly increases the Cas9-induced mutation rate in wheat using our Agrobacterium-mediated gene editing protocol. Indeed, in a previous study, exposing T_1_ transgenic wheat plants harboring the *ZmUbipro:Cas9* and *TaU6pro:sgRNA* expression cassettes to 28°C or 37°C increased the mutation rate compared to transgenic plants treated at 20°C (Milner et al. 2020). T_1_ wheat plants harboring the *ZmUbipro:Cas9* transgene showed a higher mutation rate after two heat shock treatments at 37°C for 24 h with a 16°C interval for 24 h compared to treatment at 16°C–18°C (Xu et al. 2022). Heat treatment at 34°C for 24 h also increased the Cas9-induced mutation rate in immature wheat embryos when applied just after particle bombardment (Tanaka et al. 2022). Therefore, including a heat treatment in the transformation protocol and in the resulting progeny could help increase the mutation rate to enhance the effectiveness of genome editing.

The most likely reason for the increased mutation rate after heat treatment is an increase in Cas9 activity. Cas9 nuclease activity was shown to be higher at 37°C than at 22°C in vitro (Kurokawa et al. 2021). The *ZmUbi* promoter was more active at 37°C than at 22°C in transgenic wheat plants (Milner et al. 2020). However, we did not observe an obvious increase in *Cas9* expression after a 1-day treatment at 30°C compared to a 1-day treatment at 25°C (Supplementary Figure S2). Under our experimental conditions, inoculation with Agrobacterium would strongly affect the expression of *Cas9* and mask the effect of the temperature increase. Therefore, we believe that the increase in Cas9 nuclease activity itself affects the Cas9-induced mutation rate under our experimental conditions. The rate of variation of mutated sequences also increased after a 1-day treatment at 30°C. Warm temperatures (28°C) influence the accumulation of mutations in Arabidopsis (Lu et al. 2021). High temperatures may restrict the normal DNA-repair system, favoring the induction of mutations.

### No relationship is detected between the number of transgene integration sites and Cas9-induced mutation rates

To segregate out transgenes from genome-edited crops, it is important to consider the number of transgene integration sites. We previously obtained genome-edited plants when targeting *TaQsd1*; three genome-edited plants were estimated to have four, five, and more than seven integration sites, whereas five non-edited plants were estimated to have one, two, and more than three integration sites (Abe et al. 2019). We speculated that a higher number of transgene integration sites would cause an increase in the number of mutated homoeologs.

In the current study, we explored this possibility by analyzing the segregation ratios of the transgene(s) in the progeny seedlings by PCR, from which we estimated the number of integration sites in the founder T_0_ plant. We estimated that the original T_0_ plant contained one integration site for measured segregation ratios between PCR-positive and PCR-negative seedlings of 9 : 7, 10 : 6, 11 : 5, 12 : 4, and 13 : 3 in the T_1_ progeny. Similarly, for segregation ratios of 14 : 2, 15 : 1, and 16 : 0, we estimated that the original T_0_ plant contained one or two, two, and three or more integration sites, respectively. We classified all transformants as a function of the number of mutations in the corresponding T_0_ plants and the estimated number of integration sites (Table 3). In the experiment using the TQ1_t1/b vector under the “25°C” condition, we obtained one T_0_ plant (T7) with one estimated transgene integration site and one mutation. However, three T_0_ plants produced at “25°C” (T6, T8, T20) and two T_0_ plants obtained at “30-1d” (T1, T14) harbored more than two integration sites but had no mutations. These results suggest that increasing the number of transgene integration sites does not directly result in an increased rate of Cas9-induced mutations.

**Table 3:** Table 3. Transgene integration sites, as estimated based on the segregation ratios in the progenies.

Condition	Number of mutations in T_0_ plants	Segregation of the transgene in T_1_ plants (+ : −)	Segregation of the transgene in F_1_ plants (+ : −)	Estimated number of transgene integration sites	Plant line number
*OsU6*, “25°C”	0	9 : 7–13 : 3	NA	1	O3–O6, O9, O14, O21
		14 : 2	NA	1–2	O11, O15
		15 : 1	NA	2	O25
		16 : 0	NA	>2	O10, O26
*OsU6*, “30-1d”	0	11 : 5–13 : 3	NA	1	O8, O18, O19
		14 : 2	NA	1–2	O12, O17
		16 : 0	NA	>2	O1, O2, O7, O13, O16, O22–O24
	1	13 : 3	NA	1	O20
*TaU6*, “25°C”	0	12 : 4–13 : 3	NA	1	T3, T21
		14 : 2	NA	1–2	T17, T25
		15 : 1	NA	2	T5
		16 : 0	NA	>2	T6, T8, T20
	1	13 : 3	NA	1	T7
		15 : 1	NA	2	T18
	3	12 : 4	NA	1	T19
	6	16 : 0	NA	>2	T12, T26
*TaU6*, “30-1d”	0	11 : 5–13 : 3	NA	1	T4, T11, T22
		14 : 2	NA	1–2	T9, T13
		16 : 0	NA	>2	T1, T14
	2	16 : 0	NA	>2	T15
	4	16 : 0	NA	>2	T2
	6	16 : 0	21 : 3	3	T24
		16 : 0	24 : 0	>4	T16, T23
		no seeds		NA	T10

NA, not applicable.

However, the results of segregation analysis raised the possibility that heat treatment increased the number of integration sites. Under the “25°C” condition, we isolated eleven T_0_ plants (seven for OQ1_t1/b and four for TQ1_t1/b) with one transgene integration site and seven T_0_ plants (two for OQ1_t1/b and five for TQ1_t1/b) with more than three transgene integration sites. Under the “30-1d” condition, we obtained four T_0_ plants (four for OQ1_t1/b and zero for TQ1_t1/b) with one transgene integration site and 15 T_0_ plants (eight for OQ1_t1/b and seven for TQ1_t1/b) with more than three transgene integration sites. We analyzed the segregation ratios using the F_1_ progeny of three T_0_ plants with six mutations produced under the “30-1d” condition and estimated the number of integration sites in the T_0_ plant (Table 3). We estimated one line (T24) to contain three integration sites, and the two other lines (T16, T23) contained more than four integration sites. These results indicate that increased numbers of integration sites can occur due to rising temperatures. However, under moderate heat treatment, we obtained transformants with six mutations in *TaQsd1* and only a small number of integration sites.

## Conclusion

Here, we showed that the use of the *TaU6* promoter and heat treatment at 30°C for 1 day improved the genome editing efficiency of wheat via Agrobacterium-mediated transformation. Our preliminary experiment suggested that the regeneration of wheat plants is inhibited by 37°C heat treatment. Heat treatment at 30°C for 1 day increased the Cas9-induced mutation rate, the variation in mutation types, and the number of transgene integration sites. However, we obtained genome-edited plants with mutations in all six copies of *TaQsd1* with a small number of integration site using the *TaU6* promoter and 1 day of heat treatment at 30°C. To generate genome-edited wheat, increasing the Cas9-induced mutation rate is preferred, but the regeneration rate is important and an increase in the number of transgene integration sites should be considered as well. Our results indicate that the nature of the temperature treatment is an important factor in improving the mutation rate of genome editing, with 30°C for 1 day providing an adequate increase in this rate. It will be important to evaluate the temperature conditions used in the transformation protocol to further expand the utility of genome editing.

## Abbreviations

*bar*: *bialaphos resistance gene*
CAPS: cleaved amplified polymorphic sequence
Cas9: CRISPR-associated protein 9
CRISPR: clustered regularly interspaced short palindromic repeats
*nos*: *nopaline synthase*
*OsU6* pro: *U6* promoter from *Oryza sativa*
*Qsd1*: *quantitative trait locus for seed dormancy 1*
sgRNA: single guide RNA
*TaU6* pro: *U6* promoter from *Triticum aestivum*
*ZmUbi* pro: *Zea mays Ubiquitin* promoter

## Description of Supplementary Files

Supplementary tables and figures are available online.
