# Supplementary material for: Optimizing genome editing efficiency in wheat: Effects of heat treatments and different promoters for single guide RNA expression
Source: Plant Biotechnol (Tokyo). 2023 Sep 25;40(3):237–45. doi: 10.5511/plantbiotechnology.23.0717a (PMC10901157; doi:10.5511/plantbiotechnology.23.0717a)
Supplement: Supplementary Data [file plantbiotechnology-40-3-23.0717a-s001.pdf]

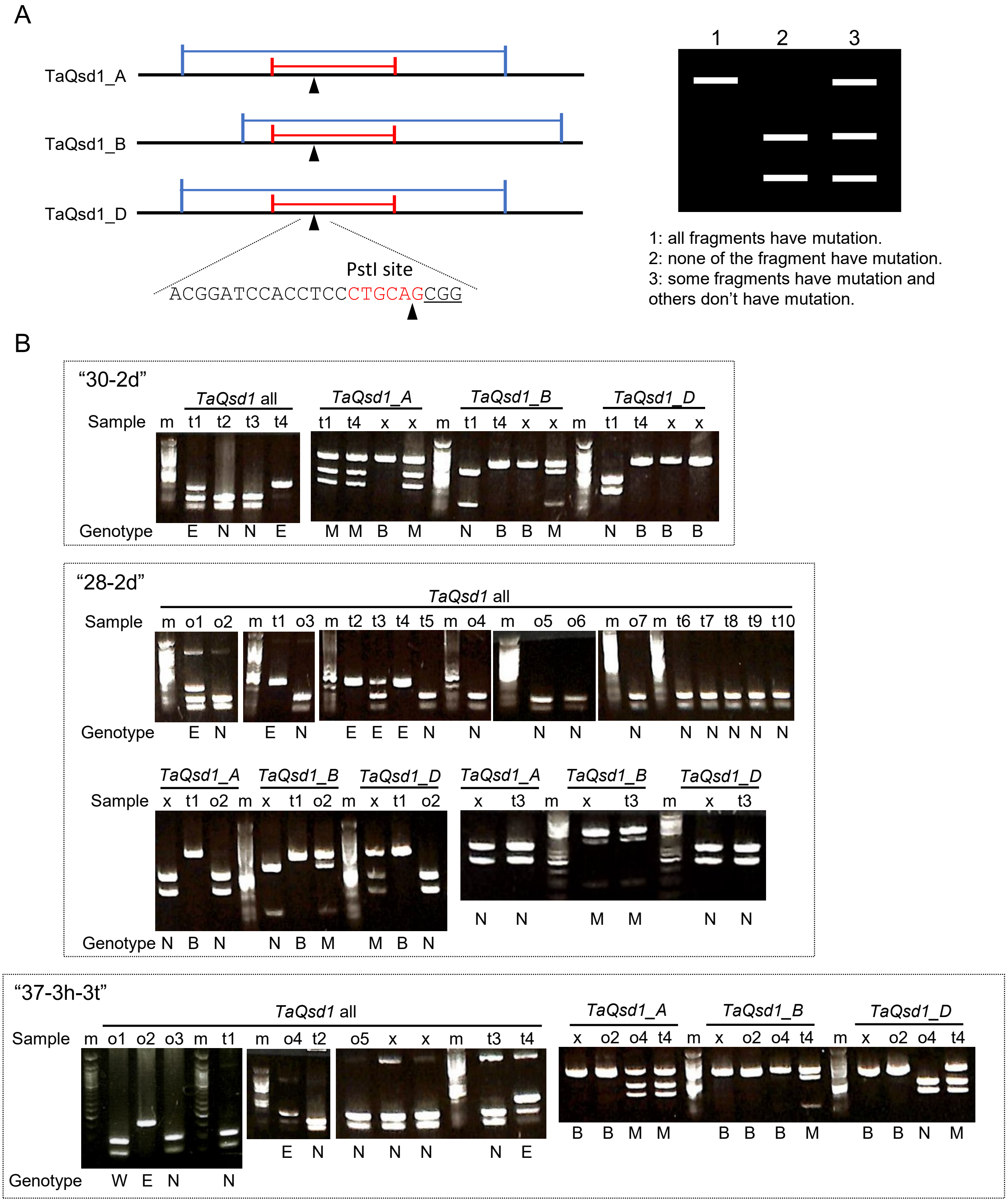

Supplementary Figure S1. CAPS analysis to determine *TaQsd1* genotype in preliminary experiments.

A. Diagrams of DNA fragment length from CAPS analysis (left panel). Red bars indicate the position of DNA fragments for *TaQsd1* all, which include all *TaQsd1* copies. Blue bars indicate the position of DNA fragments for specific *TaQsd1* copies on each subgenome. Triangles indicate the cut site of restriction enzyme PstI. In CAPS analysis, PCR products were treated with PstI and analyzed their length by electrophoresis. A diagram of electrophoresis pattern is indicated in right panel.

B. Result of CAPS analysis in preliminary experiments. t1-t10 and o1-o7 indicate the line number of regenerated plants. m: DNA size marker. x: samples from another experiment. E: part of *TaQsd1* copies were edited. N: none of *TaQsd1* copy was edited. B: Bi-allelic mutation on each subgenome. M: mono-allelic mutation on each subgenome.

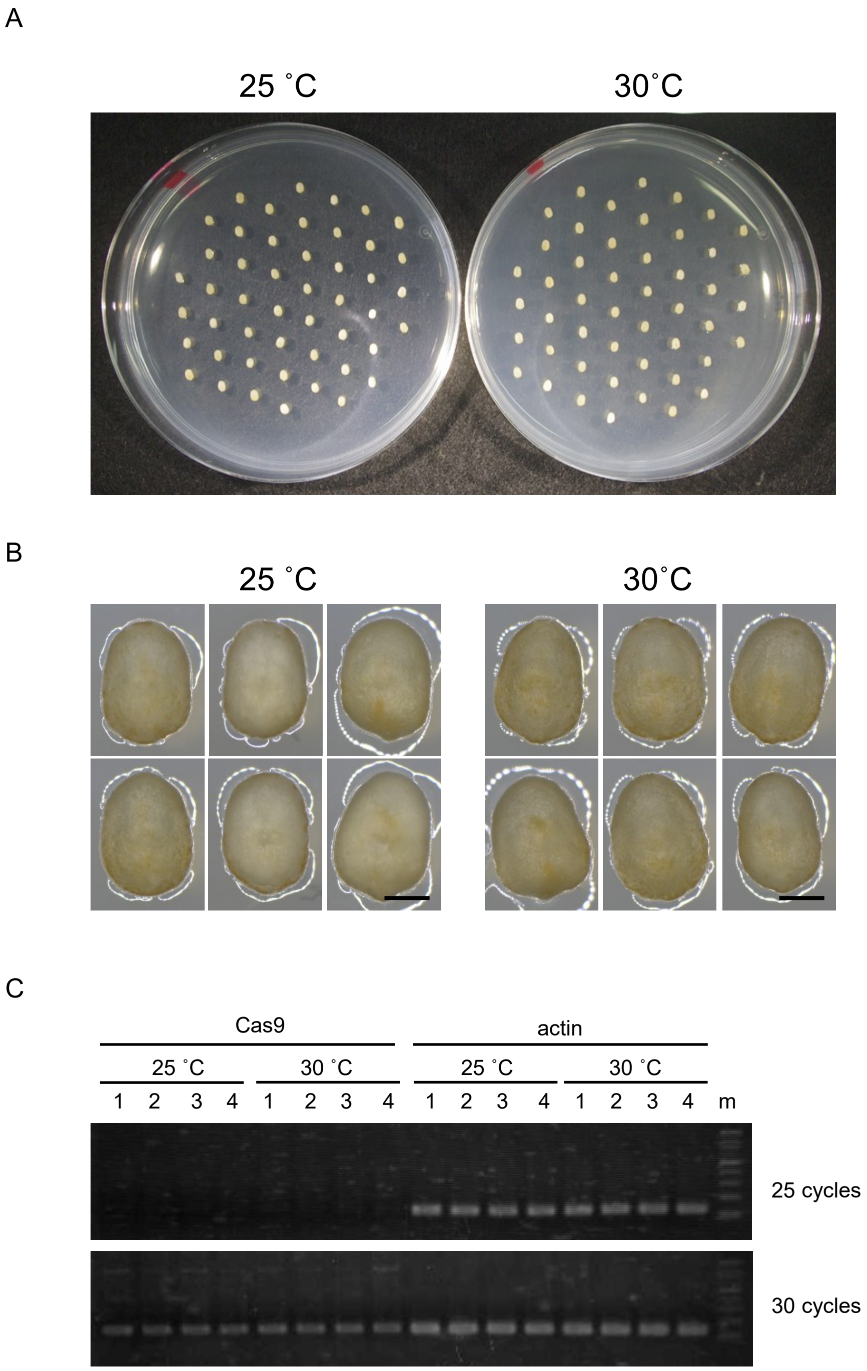

Supplementary Figure S2. Comparison of the physical appearance and *Cas9* expression level in immature embryos after heat treatment.

Immature embryos were inoculated with *Agrobacterium* harboring TQ1_t1/b vector and incubated at 25 ° C or 30 ° C for 1 day.

A. Photograph of immature embryos at resting stage after 30 ºC (right plate) or 25 ºC (left plate) for one day with the TQ1_t1/b vector.

B. Photograph of each immature embryos using stereoscopic microscope. Scale bars indicate 1 mm length.

C. RT-PCR analysis of *Cas9* and *actin* genes in immature embryos after one day heat treatment. Four independent sample with ten immature embryos were used. m: DNA size marker.

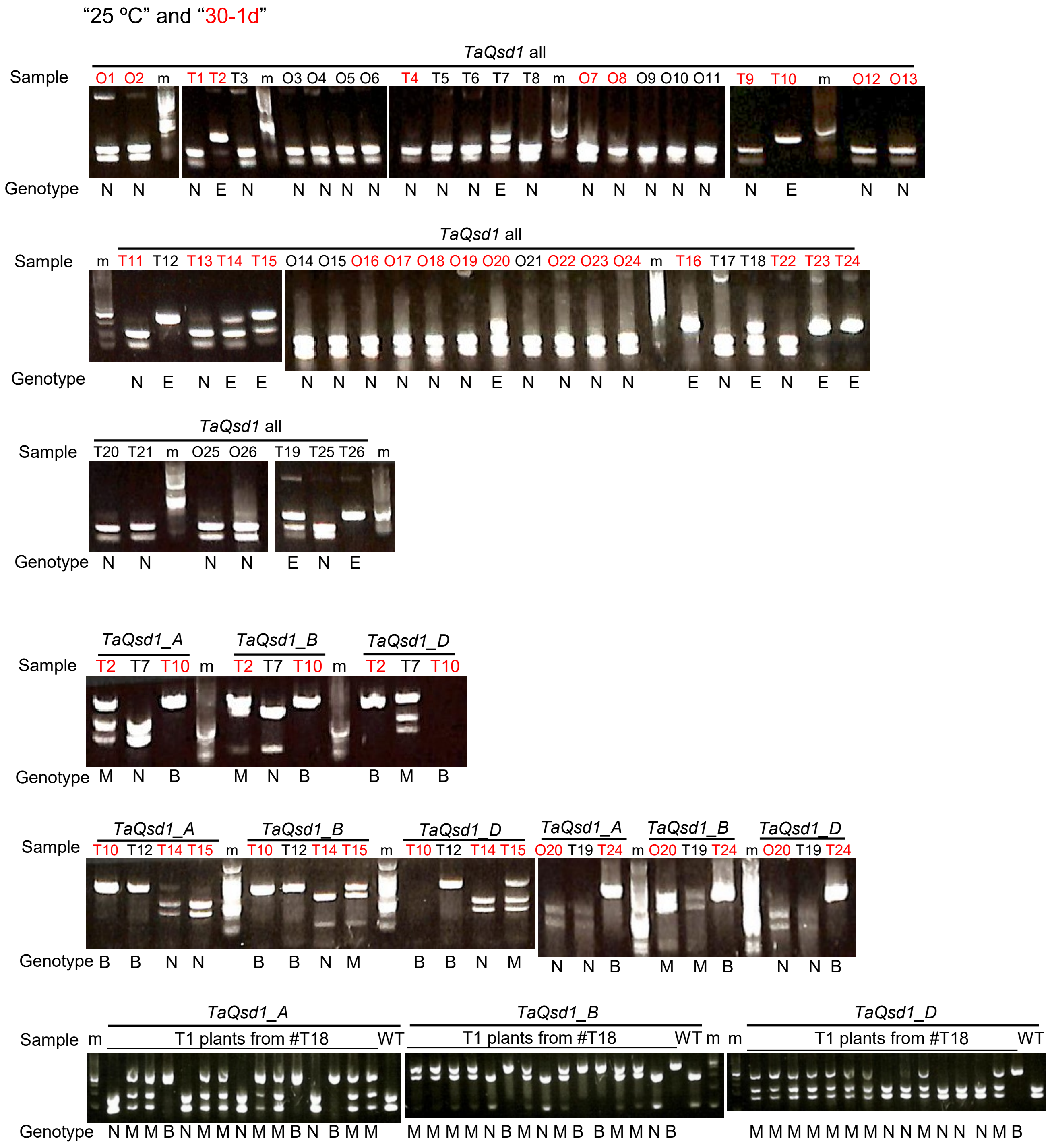

Supplementary Figure S3. CAPS analysis to determine *TaQsd1* genotype in heat treatment. T1-T26 and O1-O26 indicate the number of regenerated plants. m: DNA size marker. E: part of *TaQsd1* copies were edited. N: none of *TaQsd1* copy was edited. B: Bi-allelic mutation on each subgenome. M: mono-allelic mutation on each subgenome.

T10 plant did not showed TaQsd1_D DNA fragment and were thought to have large deletion. Mutation type of T18 plant was determined using T_1 plants. From CAPS analysis on all TaQsd1 copies, T12, T16, T23 and T26 plants were considered as *aabbdd* mutants and analyzed their mutation sequence using T_1 plants.

A

```
Qsd1_t1               ACGGATCCACCTCCCTGCAG
Off_target_candidate  ATAGATTCACCTCCCTGCAG
```

B

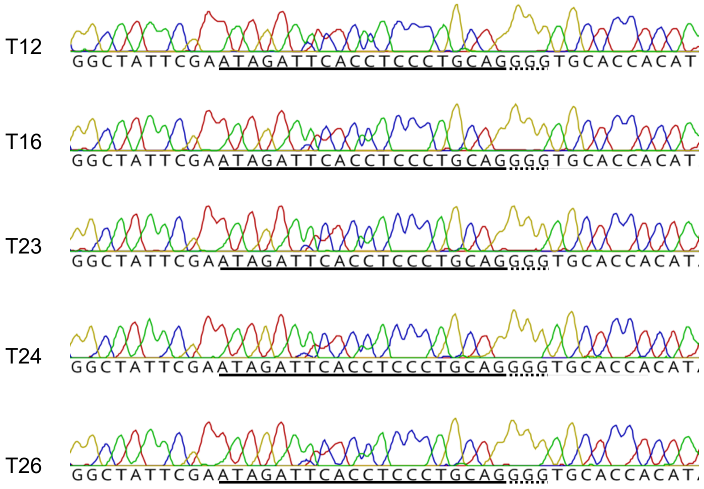

Supplementary Figure S4. Sequence analysis of *TaQsd1* t1 off-target candidate site.

A. Sequence of *TaQsd1* t1 site and its off-target candidate site.

B. Sequencing chromatogram data of off-target candidate site. DNA samples were obtained from T1 seedlings of taqsd1 triple mutant. Solid lines indicate sequences corresponding off-target candidate sequence. Dotted lines indicate positions of possible PAM sequence.

Supplementary Table S1. Oligonucleotides used in this work.

Target	analysis	oligonucreotide sequence (from 5' to 3')
Cas9	detection of transgene	CAATAGTAGGTTCGCCTGGATG
	expression analysis	TTCGTTGGGGAGGTTCTTG
bar	detection of transgene	GGTCTGCACCATCGTCAACC
		GTCATGCCAGTTCCCGTGCT
TaQsd1, all	CAPS	CAGCCTGGAGGGAATGACC
		ACCTGGTGGAATCCAGAGC
TaQsd1, A genome specific	CAPS	CACATTGTCAACAAGCACACCA
	sequence analysis	GGAGCAAAATGAGTGAATCCGTA
TaQsd1, B genome specific	CAPS	CTGGCCCTCATGTGGTCTTC
	sequence analysis	GGGATCATCGCCTTGATCTTG
TaQsd1, D genome specific	CAPS	CATACGCACTGCCTCCTTTTCA
	sequence analysis	GTTTCGCCCAGACACCTTTGTT
ZmUbipro-bar-nosT	construction	TAGATATGAGGGTCCTGCCAAGCTTGCATGCCTG
		CACTATGGTCGACCTCTATGACATGATTACGAATTCCCG
pZH_gYSA_PubiMMCas9	construction	AGGTCGACCATAGTGACTGGATA
		GGACCCTCATATCTATACCCT
Actin	expression analysis	CTATGTTCCCGGGTATTGCT
		AAGGGAGGCAAGAATCGAC
Off-target candidate of TaQsd1	1st PCR	GTTGGACCACGTAACGCAAC
		GGGAAAAGGGGGGAGCAAAG
	2nd PCR	TTGTGTCTAGGTAGGGCTGC
		TCCATCTTGCAGCAGGAGAA
	sequence analysis	CACCATGTAGCATCGTGTAGC

Supplementary Table S2. Note of each experiment for preliminary transformation (Summary in Table 1).							
Experiment number	Heat treatment	sgRNA promoter	Number of embryos	Number of transgenic plants	Plant line number	Number of genome-edited plants	mutant genotype and plant number
A18-6	30°C, 2 d (30-2d)	OsU6	50	0		0	0
A18-6	30°C, 2 d (30-2d)	TaU6	60	4	30-2d_t1, t2, t3, t4	2	AaBBDD, 30-2d_t1<br>Aabbdd, 30-2d_t4
A18-12	28°C, 2 d (28-2d)	OsU6	55	3	28-2d_o1, o2, o3	0	
A18-12	28°C, 2 d (28-2d)	TaU6	71	1	28-2d_t1	1	aabbdd, 28-2d_t1
A18-16	28°C, 2 d (28-2d)	OsU6	68	1	28-2d_o4	0	
A18-16	28°C, 2 d (28-2d)	TaU6	67	4	28-2d_t2, t3, t4	3	aabbdd, 28-2d_t2, t4<br>AABbDD, 28-2d_t3
A18-20	28°C, 2 d (28-2d)	OsU6	69	2	28-2d_o5, o6	0	
A18-20	28°C, 2 d (28-2d)	TaU6	66	0		0	
A18-28	28°C, 2 d (28-2d)	OsU6	57	1	28-2d_o7	0	
A18-28	28°C, 2 d (28-2d)	TaU6	67	5	28-2d_t5, t6, t7, t8, t9	0	
A18-48	37°C, 3 h/d × 3	OsU6	72	0		0	
A18-48	37°C, 3 h/d × 3	TaU6	73	0		0	
A18-50	37°C, 3 h/d × 3	OsU6	70	0		0	
A18-50	37°C, 3 h/d × 3	TaU6	75	0		0	
A18-52	37°C, 3 h/d × 3 (37-3hx3)	OsU6	73	1	37-3hx3_o1	0	
A18-52	37°C, 3 h/d × 3	TaU6	70	0		0	
A18-54	37°C, 3 h/d × 3	OsU6	75	0		0	
A18-54	37°C, 3 h/d × 3	TaU6	69	0		0	
A18-56	37°C, 3 h/d × 3 (37-3hx3)	OsU6	64	2	37-3hx3_o2, o3	1	aabbdd, 37-3hx3_o2
A18-56	37°C, 3 h/d × 3	TaU6	69	1	37-3hx3_t1	0	
A18-58	37°C, 3 h/d × 3	OsU6	74	0		0	
A18-58	37°C, 3 h/d × 3	TaU6	65	1	37-3hx3_t2	0	
A18-60	37°C, 3 h/d × 3 (37-3hx3)	OsU6	65	1	37-3hx3_o4	1	AabbDD, 37-3hx3_o4
A18-60	37°C, 3 h/d × 3	TaU6	63	0		0	
A18-66	37°C, 3 h/d × 3	OsU6	74	0		0	
A18-66	37°C, 3 h/d × 3 (37-3hx3)	TaU6	70	2	37-3hx3_t3, t4	1	AaBbDd, 37-3hx3_t4

Supplementary Table S3. Note of transformation experiments to analyze the effect of heat treatment (Summary in Table 2).								
Experiment number	Heat treatment	sgRNA promoter	Number of embryos	Number of transgenic plants	Plant line number	Number of genome-edited plants	mutant genotype and plant number	Mutation type of each TaQsd1 copies in taqsd1 triple mutants
A18-68	30°C, 1 d (30-1d)	TaU6	72	2	T1, T2	1	AaBbdd, T2	
A18-68	30°C, 1 d (30-1d)	OsU6	72	2	O1, O2	0		
A18-70	25°C	OsU6	66	4	O3-O6	0		
A18-70	25°C	TaU6	65	1	T3	0		
A18-72	30°C, 1 d (30-1d)	TaU6	68	1	T4	0		
A18-72	30°C, 1 d (30-1d)	OsU6	79	2	O7, O8	0		
A18-74	25°C	OsU6	60	3	O9-O11	0		
A18-74	25°C	TaU6	65	4	T5-8	1	AABBDd, T7	
A18-76	30°C, 1 d (30-1d)	TaU6	73	3	T9-T11	1	aabbdd, T10	ND (no T1 seed was obtained)
A18-76	30°C, 1 d (30-1d)	OsU6	72	2	O12, O13	0		
A18-78	25°C	TaU6	72	1	T12	1	aabbdd, T12	A (-4bp, homo allelic), B (+1bp(A), homo allelic), D (+1bp(G), homo allelic), 3 types
A18-78	25°C	OsU6	79	0		0		
A18-80	30°C, 1 d (30-1d)	OsU6	74	0		0		
A18-80	30°C, 1 d (30-1d)	TaU6	79	3	T13-T15	1	AABbDd, T15	
A18-82	25°C	OsU6	76	0		0		
A18-82	25°C	TaU6	76	0		0		
A18-84	30°C, 1 d (30-1d)	TaU6	71	0		0		
A18-84	30°C, 1 d (30-1d)	OsU6	74	0		0		
A18-86	25°C	TaU6	76	0		0		
A18-86	25°C	OsU6	74	2	O14, O15	0		
A18-88	30°C, 1 d (30-1d)	OsU6	76	5	O16-O20	1	AABbDD, O20	
A18-88	30°C, 1 d (30-1d)	TaU6	74	1	T16	1	aabbdd, T16	A (+1bp (C), deletion(> 201bp)), B (+1bp(A), +1bp(C)), D (+1bp(A), homo allelic), 5 types
A18-90	25°C	OsU6	65	1	O21	0		
A18-90	25°C	TaU6	76	5	T17-T21	2	AABbDD, T19<br>AaBbDd, T18	
A18-91	30°C, 1 d (30-1d)	TaU6	77	3	T22-T24	2	aabbdd, T23, T24	T23: A (+1bp (C), +1bp(T)), B (+1bp (A), +1bp(C)), D (+1bp(C), +1bp(T)), 6 types<br>T24: A (+1bp (C), homo allelic), B (+1bp (T), -3bp), D (+1bp (A), -2bp/+1bp(C->T)), 5 types
A18-91	30°C, 1 d (30-1d)	OsU6	79	3	O22-O24	0		
A18-94	25°C	TaU6	79	0		0		
A18-94	25°C	OsU6	70	2	O25, O26	0		
A18-96	30°C, 1 d (30-1d)	OsU6	77	0		0		
A18-96	30°C, 1 d (30-1d)	TaU6	79	0		0		
A18-98	25°C	OsU6	77	0		0		
A18-98	25°C	TaU6	77	2	T25, T26	1	aabbdd, T26	A (+1bp (A), -1bp), B (+1bp (T), homo allelic), D (+1bp (T), -2bp), 5 types
